# Inhibition of homologous recombination repair with Pentoxifylline targets G2 cells generated by radiotherapy and induces major enhancements of the toxicity of cisplatin and melphalan given after irradiation

**DOI:** 10.1186/1748-717X-1-12

**Published:** 2006-05-03

**Authors:** Lothar Bohm

**Affiliations:** 1 Department of Pharmacology, University of Pretoria P.O. Box 2034, Pretoria 0001, South Africa

## Abstract

The presentation reviews the modus operandi of the dose modifying drug Pentoxifylline and the dose enhancement factors which can be achieved in different cell types. Preclinical and clinical data show that Pentoxifylline improves the oxygenation of hypoxic tumours and enhances tumour control by irradiation. In vitro experiments demonstrate that Pentoxifylline also operates when oxygen is not limiting and produces dose modifying factors in the region of 1.2 – 2.0. This oxygen independent effect is poorly understood. In p53 mutant cells irradiation induces a G2 block which is abrogated by Pentoxifylline. The enhancement of cell kill observed when Pentoxifylline and irradiation are given together could arise from rapid entry of damaged tumour cells into mitosis and propagation of DNA lesions as the result of curtailment of repair time. Recovery ratios and repair experiments using CFGE after high dose irradiation demonstrate that Pentoxifylline inhibits repair directly and that curtailment of repair time is not the explanation. Use of the repair defective xrs1 and the parental repair competent CHO-K1 cell line shows that Pentoxifylline inhibits homologous recombination repair which operates predominantly in the G2 phase of the cell cycle. When irradiated cells residing in G2 phase are exposed to very low doses of cisplatin at a toxic dose of 5 %. (TC: 0.05) massive toxicity enhancements up to a factor of 80 are observed in melanoma, squamous carcinoma and prostate tumour cell lines. Enhancements of radiotoxicity seen when Pentoxifylline and radiation are applied together are small and do not exceed a factor of 2.0. The capacity of Pentoxifyline to inhibit homologous recombination repair has not as yet been clinically utilized. A suitable application could be in the treatment of cervical carcinoma where irradiation and cisplatin are standard modality. In vitro data also strongly suggest that regimes where irradiation is used in combination with alkylating drugs may also benefit.

## Introduction

The methylxanthine drug Pentoxifylline (TRENTAL, Sanofi-Aventis) is clinically well established for the treatment of cerebral ischemia and a variety of other vasoocclusive disorders such as intermittent claudication. [14,50]. Systemically the drug operates by enhancing the flexibility of blood cell membranes and reducing blood viscosity. The positive influence of Pentoxifylline on the microcirculation and peripheral oxygenation has led to applications in experimental radiotherapy showing small but consistent improvements of the radiotoxicity. Injection of PFX followed by irradiation 45 minutes later produces a 1.1–1.5 fold enhancement of tumour growth delay and this is associated with a marked reduction of tumour anoxia.[10,23,25-27,41,42,47,52].

Apart from the systemic effects there is now a long list of published data indicating that Pentoxifylline (and the related drug caffeine) influences the radiobiological responses of tumour cells in an oxygen independent manner. Given to tumour cell cultures before irradiation at the subtoxic dose of 2 mM, Pentoxifylline effectively enhances radiotoxicity measured by clonogenic cell survival by factors of 1.2–2.0 [3,4,9,45-47].

In the following I summarize our own [54] and other new in vitro data which explore the effects of this interesting drug on the damage responses of tumour cells. It is shown that Pentoxifylline emerges as an effective repair inhibitor and that this new mechanistic understanding shows great promise for application in tumour therapy.

## Materials and methods

The determination of the drug toxicity enhancement factors for Melphalan, Daunorubicin and Cisplatin are based on chemosensitivity measurements using the crystal violet assay. G2/M block abrogation and measurment of cell survival under conditions of G2 block abrogation and drug addition involved control experiments measuring cell survival for irradiation alone, Pentoxifylline alone, Pentoxifylline plus irradiation, Pentoxifylline plus drug at 5 % toxicity and cytostatic drug alone. Data obtained by addition of Pentoxifylline were normalized with respect to controls containing Pentoxifylline alone. 12–20 hours postirradiation when the G2 block assesed by flow cytometry was maximally expressed, Pentoxifylline at 2 mM was added. The cytostatic drugs Daunorubicin, Melphalan or Cisplatin were added at a concentration which resulted in 5 % toxicity for drug only controls. These low drug concentrations were necessary to ensure detection of the toxicity enhancements induced by Pentoxifylline in the survival and vital dye staining assays. The additions were made either at the time of maximum G2 block expression or 7 hours later when the G2 block had been abrogated. For further experimental detail consult [4].

For cytotoxicity and repair experiments the normal CHO-K1 hamster cell line and the repair deficient xrs1-mutant cell line were grown in MEM alpha medium as decribed [65]. Irradiation and cell survial by clonogenic assay were as given in [4]. Exposure to 2 mM Pentoxifylline, 1 mM Caffeine, 15 μ or 20 mM dimethylsulfoxide (DMSO) was for 40 minutes in medium before irradiation and for 20 hrs after irradiation when the medium was replaced with drug free medium and incubation continued until formation of viable colonies. Pentoxifylline toxicity was determined over a dose range of 0–8 mM, Caffeine 0–4 mM and Wortmannin 0–25 μM. Wortmannin served as inhibitor of non-homolgous endjoining (NHEJ) repair. Repair assessment by constant field gel electrophoresis (CFGE) was as previously described [45,66]. Confluent cultures were used to minimize S-phase variation. Encapsulation of cells in agarose plugs was as given previously [45,66] to avoid non-specific DNA damage. Cells harvested by trypsinisation were resuspended in a 0.5 % low melting point agarose solution and aliquots of 30 μl, containing ~0.5 × 10^5 ^cells, were placed into each well of a disposable plug mold (BioRad), and allowed to solidify at 4°C for 45 min. Plugs were irradiated in ice-cold MEM containing 2 % HEPES, over a dose range of 0–100 Gy at dose rate of 2.78 Gy/min. The residual damage was determined by incubating plugs at 37°C in growth medium for periods of 2–20 hours. For repair in the presence of Pentoxifylline or Caffeine the cells were irradiated with ^60^Co γ -irradiation in ice-cold MEM containing 2 % HEPES and 2 mM Pentoxifylline, or 1 mM Caffeine over a dose range of 0–100 Gy on ice. After irradiation the plugs were transferred to preheated (37°C) MEM alpha medium containing the same drug concentrations and incubated for 2 and 20 hours to allow for repair.

For both protocols (initial and residual damage), plugs were submersed in an ice-cold lysing solution containing 50 mM EDTA, 1 % N-lauryl-sarcosine and 1 mg/ml Proteinase K. Incubation of 1 hour at 4°C was followed by lysing at 37°C for 20 hours. Agarose plugs were then washed five times (50 mM EDTA) and stored in 2 ml of 50 mM EDTA solution. Agarose plugs were loaded into a 20 × 20 cm 0.6 % agarose gel and run in 0.5 × TBE buffer for 30 hours at a constant field strength of 1.2 V/cm. Gels were stained with ethidium bromide (0.5 μg/ml in 0.5 × TBE) and subjected to fluorometric analysis with a GeneSnap (VacuTec) image analysis system. The fraction of DNA released from the plug was obtained from the following equation:



where fl_rel _and fl_plug _correspond to fluorescence measured in the lane with high mobility DNA and non-mobile DNA in the control plug respectively. Untreated control samples were used for each sample subset to subtract background fluorescence caused by non-specific DNA degradation. Dose response curves were obtained by plotting dose (Gy) vs. the fraction of DNA released (F_rel_) as calculated above, representing initial damage (0 hours), residual damage (2 hours) and residual damage (20 hours). Since data could not be fitted by linear regression, data points were connected and the area under the curve (AUC) was calculated for each curve in the GraphPad Prism (GraphPad software, San Diago, USA) computer program.

## Results and discussion

### Enhancement of radiotoxicity is tumour cell specific

The capacity of Pentoxifylline to enhance cytotoxicity was first noted in T24 bladder tumour cells treated with the alkylating agent Thiotepa showing a 3 fold reduction of cell survival and stimulation of mitotic progression [17]. Using Cobalt 60 irradiation as the intervention and SF2 as the reference point we demonstrated that 2 mM Pentoxifylline enhances the radiotoxicity by a factor of 1.10 in V79 cells and by a factor of 1.60 in Hela cells [47]. The radiotoxicity enhancement factors are cell line and irradiation dose dependent and range from 1.2 to 2.0 at 2 Gy reaching 3–14 at 10 Gy in human melanoma, squamous cell carcinoma and in murine cell lines [4,5,45]. Caffeine at 1 mM has been found to enhance radiotoxicity by margins of 1.4 – 4.6 in US9-93 and by 1.4–2.0 in LMS6-93 human sarcoma lines [2]. 1 mM caffeine and 2 mM Pentoxifylline alone are not cytotoxic in most cell lines [4,7]. A trend first noted in A 549 p53 transfectants was the observation that radiosensitisation by methylxanthines operates more effectively in p53 mutant than in p 53 wild type cells [22,39]. This has since been corroborated in a wide range of p53 wild type and p53 mutant pairs of cell lines [2,4,5,40]. The sensitizing influence of Pentoxifylline and Caffeine is not restricted to irradiation but also applies to cytotoxic drugs [2,17,22,29].and to UV-irradiation [11,31].

### Abrogation of G2 blocks and indications for a role of repair

Evidence for a role of Pentoxifylline and caffeine in repair comes from the observation that p53 mutant and p53 defective cells in general are preferentially sensitized to irradiation whereas p53 wild type cells show no or only a mild sensitisation response [15,38,47]. These circumstances would offer a mechanism for selective targeting of tumour cells [15,19,22,39]. In this mechanism irradiated cells blocked in G2 phase would progress prematurely into mitosis and G1 phase and thus be subjected to a shortened repair time [28,37,39]. We have shown that the abrogating effect involves early restoration of cyclin B1 and p34 cdc-2 levels in the mitosis promoting factor to pre-irradiation levels [43,44] and also gives rise to a 3–5 fold increase of Histone H3 phosphorylation [3] which is diagnostic of mitotic progression [18]. When we induced the G2 M block with the spindle inhibitor Nocodazole, Pentoxifylline was ineffective in abrogating G2 blocks, leaving G2 populations in p53 mutants at the level of 80 % for 10 hrs after drug addition whereas presence of Pentoxifylline for 7 hours in irradiated cells would reduce the G 2 population from 60 to 19 % [7]. This demonstrates that Pentoxifylline does not operate at the spindle assembly checkpoint [7]. A popular explanantion for the enhanced radiotoxicity generated by Pentoxifylline has been that early transit of cells into mitosis would shorten repair time [11,15,30,37-39]. But this view may be simplistic and has been questioned by us [5,45] and by other investigators [19,35]. We have shown that addition of Pentoxifylline 12 hours postirradiation when the G2 block is maximally expressed and repair is essentially complete does not produce any radiosensitization [5,45]. The lack of a correlation between G2 block abrogation and cytotoxicity enhancement also argues against a role of reduced repair time [36]. It therefore seems that premature entry of G2 blocked cells into mitosis is not the critical event which can explain radiosensitization [5,45]. Nevertheless the conclusion that Pentoxifylline suppresses DNA repair is not without substance as shown by delayed plating experiments indicating inhibition of potential lethal damage repair [31]. Presence of 2 mM Caffeine in irradiated preimplantation chick embryo blastomeres and analysis by comet assay has shown that the restoration of DNA damage is inhibited in the early 2 hour time frame [33]. Further evidence for a role of early repair events comes from our laboratory showing that Pentoxifylline reduces the recovery ratios in 3 hour split dose experiments from 3.0 ± 0.40 to 1.0 ± 0.18 in irradiated Hela cells [47].

### New evidence supporting a role of Pentoxifylline in repair inhibition

**Figure 1:**
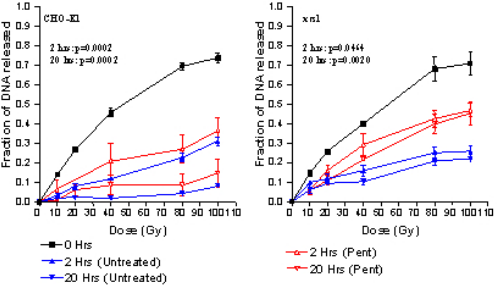
Constant field gel electrophoresis (CFGE) repair assay showing influence of Pentoxifylline on fractions of DNA released in response to irradiation dose for the parental CHO-K1 cells and NHEJ-defective xrs1 mutant cell line. Adapted from [6].

The requirement of methylxanthine presence at the time of irradiation for any sizable radiosensitization to occur [4,45] and delayed plating experiments [35] strongly suggest that early damage responses are influenced. When we evaluated DNA repair by CFGE and assessed mobile DNA fragments after high dose exposure we found unequivocal evidence that Pentoxifylline effectively suppresses DNA double strand break repair in p53 mutant human melanoma and squamous cell carcinoma lines [45] and various p53 transfectants [5]. Further significant progress in the understanding of the mechanism of action of caffeine in radiosensitization has come from experiments using repair deficient mutants. It was observed that a rad 51 paralogue XRCC2 irs1 line defective in homologous recombination (HR) shows significantly diminished caffeine radiosensitization which can be restored by expression of XRCC2 [1]. Consistent with these results is the finding that an irs-20 cell line mutant for DNA-PK and defective in non-homologous endjoining (NHEJ) is radiosensitized by caffeine to an extent comparable with wildtype cells [49]. These observations have been confirmed in other rad 51 mutants and in ATM -/- cells strongly suggesting that caffeine selectively targets steps in DNA double strand break repair which require HR [48]. Strong evidence for a role of Pentoxifylline in HR is presented in CFGE repair assays using the parental CHO-K1 cells and the NHEJ defective xrs-1 mutant. In CHO-K1 wildtype cells operating both repair pathways we found Wortmannin to strongly enhance radiotoxicity and suppress survival whereas the xrs-1 mutant that is defective in NHEJ repair showed no change of cell survival. CFGE repair data after high doses of irradiation furthermore indicated Wortmannin to inhibit repair only in the CHO-K1 wildtype cells confirming that the cell pair is a representative model for addressing the question of HR repair (Roos & Bohm, unpublished). Testing of Pentoxifylline in this system and analysis of mobile unrepaired DNA fragments shows that repair is most strongly inhibited in the xrs-1 cell line which essentially operates HR-repair only, but less strongly in the parental fully repair competent CHO-K1 cell line which also operates NHEJ repair (Figure 1). These results are in excellent agreement with data on NHEJ deficient 180BRM and XR-1 cells where caffeine induced depression of survival was found to be much greater than in the parental NHEJ proficient MRC5SV1 and CHO cell lines [49] suggesting that caffeine induced radiosensitisation is caused by an NEHJ independent process [48]. Experiments in A 549 lung adenocarcinoma and in K562 erythroblastoid leukaemia cells have shown that caffeine inhibits ATM and ATR kinases which control checkpoints via phosphorylation of p53 thereby maintaining the G2 block and controlling HR repair [21,24,32,48,51]. The close structural and functional relationship between Caffeine and Pentoxifylline strongly suggests that ATM inhibition also applies to Pentoxifylline.

Another indication that HR repair processes are extremely sensitive to Pentoxifylline is shown in experiments in which p53 mutant cells blocked in G2 by irradiation were exposed to a second cytotoxin at a dose which would induce 95 % survival or 5 % cell kill (TD05) in fully cycling cells in the absence of Pentoxifylline. Addition of such low concentrations of common cytotoxins to G2 blocked cells in the presence of Pentoxifylline generates very large enhancements of drug toxicity in the range of 2.3–2.8; 8.6–85 and 52–74 for Vinblastine, Melphalan and cisplatin respectively (Table 1). In similar experiments in p53 wildtype LNCaP and p53 mutant DU-145 and BM 12604 prostate cells we found dose enhancement factors of 1.5–4.5 for the p53 mutant cells and 1.4–1.6 for the p53 wildtype LNCaP cells Table 1 and [40]. The extreme sensitivity of repair events in G2 is evident by the fact that up to 85 fold toxicity enhancement is achieved at drug doses which are 1–3 orders of magnitude lower than standard therapeutic doses (Table 1). The wide variation of the dose enhancement between cell types and cytotoxic drugs would support a model in which repairability of the lesion plays a role.

**Table 1:** Drug toxicity enhancement factors (EF's) obtained in human tumour cell lines when cells were blocked in G2 with irradiation and then subjected to Pentoxifylline and a TC: 05 dose of a cytotoxic drug. Data adapted from [6]

Cell line ^a^		Drug	EF's		
4197	p53 wt ^b^	D, M, CP	1.3	3.0	2.6
4451	p53 mut ^b^	D, M, CP	2.3	**8.6**	**52**
Be11	p53 wt^b^	D, M, CP	1.4	2.3	1.2
MeWo	p53 mut ^b^	D, M, CP	2.8	**85**	**74**
DU 145	p53 mut ^c^	V, E, CP	**4.8**	1.5	**4.1**
BM1604	p53 mut ^c^	V, E, CP	2.6	1.0	**4.5**
LNCaP	p53 wt ^c^	V, E, CP	1.4	1.5	1.6

^a^) 4197 and 4451 are human squamous carcinoma cell lines. Be-11 and MeWo are human melanoma cell lines. The DU-145 prostatic tumour cell line was established from a metastatic central nervous system lesion. The BM 1604 cell was established from a radical prostatectomy biopsy. The LnCaP cell line was established from a supraclavicular lymph node metatstasis of a human prostate adenocarcinoma.

D: Daunorubicin; E: Etoposide; M: Melphalan; V: Vinblastine; CP: cisplatin based on clonogenic SF 7 (survival fraction at 7 Gy) and dye staining data ^b) ^Binder et al. [4] ; ^c) ^Serafin et al. [40]

### Clinical ramifications

The clinical merits of Pentoxifylline on tumour control and survival when combined with irradiation cannot as yet be judged with any finality. For stage I, II, and III non- small cell lung cancer giving 3 × 400 mg Pentoxifylline/day with conventional dose fractionation the complete and partial responses were similar but the median time to relapse was 2 month longer in the Pentoxifylline group than in controls. In the Pentoxifylline group the median survival was 18 month as compared to 7 month in the control group. The differences in 1-year and 2-year survival found to be 60 % as compared to 35 % and at 18 % as compared to 12 % respectively but were not statistically significant. This stage III randomised multicenter trial concluded that Pentoxifylline is a modestly effective radiation response modifier [57]. In a phase II evaluation on brain metastases in 14 patients the median survival time was 33 days and identical to historical controls showing no drug toxicity and supporting higher doses for future trials [58]. In another phase II single center trial on 11 patients with glioblastoma multiforme evaluating the influence of Pentoxifylline on utilization and effectiveness of cytotoxic drugs the median survival for all patients was 26 weeks from initial diagnosis showing no obvious benefits [59]. In a rhabdomyosarcoma rat model giving 50 mg Pentoxifylline/kg before irradiation we demonstrated a marked improvement of tumour oxygenation and dose enhancement factors of 1.10 for conventional dose fractionation and 1.37 for continuous hyperfractionated irradiation, the higher value perhaps arising from lower vascular injuries [52]. In the light of these data and the well-documented effectiveness of Pentoxifylline on the microcirculation it is surprising that the improvements to radiation toxicity and tumour control have remained small.

An area that now attracts great clinical interest is the ability of Pentoxifylline to ameliorate late radiation injury. Pentoxifylline inhibits the hypoxia induced upregulation of Tissue Factor (TF) a procoagulant stimulus known to upregulate VEGF and angiogenesis [60]. Pentoxifylline is also an effective phosphodiesterase inhibitor showing anti-inflammatory and immunosuppressive effects due to suppression of TNF-α and inhibition of IL-1 and IL-2 induced lymphocyte stimulation [reviewed in [52]]. In view of this broad spectrum of activities and down regulation of cytokines it is not surprising that Pentoxifylline inhibits effects that are stimulated by radiation and tissue injury. In patients receiving radiotherapy for sqamous carcinoma of the head and neck Pentoxifylline given concurrently with irradiation has been found to suppress skin fibrosis and soft tissue necrosis [61]. Given *post factum*when late tissue damage from irradiation was already manifest a combination of Pentoxifylline and the antioxidant α -Tocopherol has been found to be effective in reducing grade I-II radiation proctitis/enteritis [62] and in the regression of superficial radiation induced fibrosis [63].

A recent clinically motivated study on T 98 G human glioma cells has demonstrated that application of Pentoxifylline before 3 intermittent irradiation doses of 4 Gy consistently sensitised cells by a factor of 4 as compared to a single dose of 12 Gy in the presence of Pentoxifylline [13]. This suggests a role for Pentoxifylline in repair inhibition between dose fractions and possible application in stereotactic surgery [13]. Another clinically realistic scenario emerges from the toxicity enhancements observed when irradiated tumour cells which have entered the G2 cell cycle block are subjected to cisplatin (Table 1). Irradiation and cisplatin are accepted modalities in the treatment of cervical cancer where low doses of cisplatin are given as a radiosensitizer. In this case cisplatin operates by mildly inhibiting NHEJ repair of irradiation induced double strand breaks [55,56]. In view of the very high in vitro toxicity enhancement factors measured in melanoma and squamous carcinoma lines (Table 1) and assuming a p53 mutant status of the tumour, introduction of Pentoxifylline and cisplatin to tumour cells blocked in G2 by irradiation, could markedly improve the therapeutic outcome. The approach has been recommended for clinical trials by an international panel assembled by the IAEA [53]. With the addition of Pentoxifylline and cisplatin to irradiated tumours it may indeed be possible to harness a very substantial cisplatin toxicity which is not seen when cisplatin is given as a radiosensitizer in the absence of Pentoxifylline. Another candidate for the combination of Pentoxifylline with a cytotoxic drug would be melphalan which shows an even higher (85 fold) increase of toxicity in G2 cells when homologous recombination is inhibited. It is interesting to note that the two drugs showing the highest toxicity enhancements, i.e. Cisplatin and Melphalan when given alone produce a lethal double strand lesion only after replication [20]. In the scenario suggested here the drugs would be given after irradiation when the target is already compromised by double strand breaks. Presence of the HR inhibitor and stimulation of mitotic progression may constitute a very adverse environment for genomic restitution to proceed. While the benefits of Pentoxifylline on tumour control when given postirradiation remain to be clinically verified very recent observations on BRCA2 deficient cells have indicated that persistence of DNA lesions (in this case arising postreplication from the inhibition Poly (ADP-ribose) polymerase) and a defunct HR pathway are attractive strategies for new and less toxic therapies of cancer [8,16]. I suggest that p53 mutant and p53 dysfunctional tumour cells could be effective targets for cytostatic intervention and repair inhibition in conjunction with radiotherapy.

## Conclusion

The influence of Pentoxifylline on the microcirculation and peripheral oxygenation has generated a long-standing and enthusiastic interest in applying this drug for the treatment of cancer by irradiation. While improved tumour oxygenation has been documented, the clinical benefit of Pentoxifylline when given in conjunction with irradiation to achieve better tumour control and patient survival has been modest if not disappointing. There is now a resurge of interest in this drug for controlling late radiation morbidity. This application is based upon the ability of Pentoxifylline to suppress lymphocyte activation and cytokine mediated inflammatory and fibrotic processes. At present the successes in this area seem to be more encouraging than in the former.

A large body of *in vitro *data has shown that Pentoxifylline given in conjunction with irradiation exerts an oxygen independent effect on tumour cells by enhancing the radiotoxicity by factors of 1.2 – 2.0. The simplistic and widely accepted interpretation of this effect is that the drug abrogates the G2 block and promotes early mitosis thereby propagating DNA lesions. It is shown here that G2 block abrogation is not the explanation and that damage responses rather than the initial DNA lesion are affected. Pentoxifylline directly inhibits homologous recombination repair. We find tumour cells residing in G2 phase when challenged with a cytotoxic drug and Pentoxifylline to undergo an up to 80 fold enhancement of drug toxicity. This is in line with the compromised repair options of G2 cells. This ability of Pentoxifylline to markedly enhance drug toxicity has not yet been clinically utilized but shows considerable promise in radiotherapy. A suitable application would be in the treatment of cervical cancer where irradiation and cisplatin are standard modality.

## References

[B1] Assaad NA, Zeng Z, Guan J, Thacke J, Iliakis G (2000). Homologous recombination as a potential target for caffeine radiosensitisation in mammalian cells reduced caffeine radiosensitisation in XRSS2 and XRSCC 3 mutants. Oncogene.

[B2] Bache M, Pigorsch S, Dunst J, Wurl P, Meyer A, Bartel F, Schmidt H, Rath FW, Taubert H (2001). Loss of G2/M arrest correlates with radiosensitisation in 2 human sarcoma cell lines with mutant p53. Int J Cancer.

[B3] Binder A, Bohm L (2002). Influence of irradiation and pentoxifylline on histone H3 phosphorylation in human tumour cell lines. Cell Prolif.

[B4] Binder A, Serafin AM, Bohm L (2000). Abrogation of the G2/M phase block enhances the cytotoxicity of Danourubucin, Melphalan and cisplatin in p53 mant human tumour cells. Radiat Res.

[B5] Binder A, Theron T, Donninger H, Parker MI, Bohm L (2002). Radiosensitization and DNA repair inhibition by Pentoxifylline in NIH 3T3 p53 transfectants. Int J Radiat Biol.

[B6] Bohm L, Roos WP, Serafin AM (2003). Inhibition of DNA repair by Pentoxifylline and related methylanthine derivatives. Toxicology.

[B7] Bohm L, Theron T, Binder A (2000). Influence of Pentoxifylline, A-802710 Propentofylline and A-802715 (Hoechst) on the expression of cell cycle blocks and S-phase content after irradiation damage. Biochim Biophys Acta.

[B8] Bryant HE, Schultz N, Thomas HD, Parker KM, Flower D, Lopez E, Kyle S, Meuth M, Curtin NJ, Helleday T (2005). Specific cell killing of BRCA2-deficient tumours with inhibitors of poly(ADP-ribose)polymerase. Nature.

[B9] Busse PM, Bose SK, Jones RW, Tolmach LJ (1977). The action of caffeine on X-irradiated cells. II Synergistic lethality. Radiat Res.

[B10] Collinridge DR, Rockwell S (2000). Pentoxifylline improves the oxygenation and irradiation response of BA 1112 rat rhabdomyosarcomas and EMT 6 mouse mammary carcinomas. Int J Cancer (Radiat Oncol Invest).

[B11] De Frank JS, Tang W, Powell SN (1996). p53 null cells are more sensitive to UV-light only in the presence of caffeine. Cancer Res.

[B12] Durante M, Furusawa Y, Majiama H, Kawata T, Gotoh E (1999). Association between G2-phase block and repair of radiation induced chromosome fragments in human lymphocytes. Radiat Res.

[B13] Eley KW, Benedict SH, Chung TDK, Kavanagh BD, Broaddus WC, Schmidt-Ullrich RPK, Lin PS (2002). The effects of pentoxifylline on the survival of human glioma cells with continuous and intermittent stereotactic surgery irradiation. Int J Radiat Oncology Biol Phys.

[B14] Ely H (1994). Is Pentoxifylline the drug of the decade?. J Am Acad Dermatology.

[B15] Fan S, Smith ML, Rivet DJ, Duba D, Zhan Q, Kohn KW, Fornace AJ, O'Connor PMO (1995). Disruption of p53 sensitizes breast cancer MCF-7 cells to cisplatin and pentoxifylline. Cancer Res.

[B16] Farmer H, McCabe N, Lord CJ, Tutt ANJ, Johnson DA, Richardson TB, Santarosa M, Dillon KJ, Hickson I, Knights C, Martin NMB, Jackson SP, Smith GCM, Ashworth A (2005). Targeting the DNA repair effect in BRCA mutant cells as a therapeutic strategy. Nature.

[B17] Fingert HJ, Pu AT, Chen Z, Googe PB, Alley MC, Pardee AB (1986). In vivo and in vitro enhanced antitumour effect by Pentoxifylline in human cancer cells treated with Thiothepa. Cancer Res.

[B18] Gurley LR, D'Anna JA, Barham SS, Deaven LL, Tobey RA (1978). Histone phosphorylation and chromatin structure during mitosis in Chinese Hamster cells. Eur J Biochem.

[B19] Iliakis G, Nusse M (1983). Effects of caffeine on x-irradiated synchronous, asynchronous and plateau phase mouse ascites cells: the importance of progression through the cell cycle for caffeine enhancement of cell killing. Int J Radiat Biol.

[B20] Kaina B (2003). DNA damage-triggered apoptosis: Criticial role of DNA repair, double strand breaks, cell proliferation and signalling. Biochem Pharm.

[B21] Kastan MB, Lim DS (2000). The many substrates and functions of ATM. Nat Rev Mol Cell Biol.

[B22] Kastan MB, Onyekwere O, Sidranski D, Vogelstein B, Craig RW (1991). Participation of p53 protein in the cellular response to DNA damage. Cancer Res.

[B23] Kelleher DK, Thews O, Vaupel P (1998). Regional perfusion and oxygenation of tumours upon methylxanthine derivative administration. Int J Radiat Oncology Biol Phys.

[B24] Lavin MF (1998). Radiosensitivity and oxidative signalling in Ataxia telangiectasia: An update. Radiother Ocol.

[B25] Lee I, Kim JH, Levitt SH, Song CW (1992). Increase in tumour responses by Pentoxifylline alone or in combination with Nicotinamide. Int J Radiat Oncology Biol Phys.

[B26] Lee I, Biaglow JE, Lee J, Cho M (2000). Physiological mechanisms of radiation sensitisation by Pentoxifylline. Anticancer Research.

[B27] Lee I, Boucher Y, Demhartner TJ, Jam RK (1994). Changes in tumour blood flow oxygenation and interstitial fluid pressure induced by pentoxifylline. Br J Cancer.

[B28] Lehmann AR (1972). Effect of caffeine on DNS synthesis in mammalian cells. Biophys J.

[B29] Li Y, Sun L, Weber-Johnson K, Pashoud N, Coucke P (1999). Potentiation of cytotoxicity and radiosensitisation of 2-doxy-2'(fluoro-methylene)cytidine by pentoxifylline in vitro. Int J Cancer.

[B30] Li Y, Weber-Johnson K, Sun L, Pashoud N, Mirimanoff R, Coucke PA (1998). Effect of Pentoxifylline on radiation induced G2-phase delay and radiosensitivity of human colon and cervical cancer cells. Radiat Res.

[B31] Link CJ, Orren D, Muldoon R, Cook JA, Bohr VA (1996). Pentoxifylline inhibits gene specific repair of UV-induced damage in hamster cells. Radiat Oncol Invest.

[B32] Morrison C, Sonoda E, Takao N, Shinohara A, Yamato KI, Takeda (2000). The controlling influence of ATM in homologous recombination repair of DNA damage. EMBO-J.

[B33] Muller WU, Bauch T, Wojcik A, Bocker W, Streffer C (1996). Comet assay studies indicates that caffeine mediated increase in irradiation risk of embryos is due to inhibition of DNA repair. Mutagenesis.

[B34] Murray AW (1992). Creative blocks: cell cycle checkpoints and feedback controls. Nature.

[B35] Musk SRR (1991). Reduction of radiation induced cell cycle blocks by caffeine does not necessarily lead to increased cell killing. Radiat Res.

[B36] Musk SRR, Steel GG (1990). Override of the irradiation induced mitotic block in human tumour cells by methylxanthines and its relationship to the potentiation of cytotoxicity. Int J Radiat Biol.

[B37] O'Connor PMO (1997). Mammalian G1 and G2 phase checkpoints. Cancer Surgery.

[B38] Powell SN, de Frank JS, Connel P, Eogan M, Preffer F, Dombkowski D, Tang W, Friend S (1995). Differential sensitivities of p 53 (-) and P53 (+) cells to caffeine induced radiosensitisation and override of G2 delay. Cancer Res.

[B39] Russel KJ, Wiens LW, Demers GW, Galloway DA, Le T, Rice GC, Bianco JA, Singer JW, Groudine M (1996). Preferential radiosensitisation of G1 checkpoint deficient cells by methylxanthines. Int J Radiation Oncology Biol Phys.

[B40] Serafin AM, Binder A, Bohm L (2001). Chemosensitivity of prostatic tumour cell lines under conditions of G2 block abrogation. Urol Res.

[B41] Song CW, Hasegawa T, Kwon HC, Lyons JC, Levitt SA (1992). Increase in tumour oxygenation and radiosensitivity caused by Pentoxifylline. Radiat Res.

[B42] Song CW, Makepeace CM, Griffin RJ, Hasegawa T, Osborn JL, Choi IB, Nah BS (1994). Increase in tumour blood flow by Pentoxifylline. Int J Radiat Oncology Biol Phys.

[B43] Theron T, Bohm L (1998). Cyclin B1 expression in response to abrogation of the irradiation induced G2 block in Hela cells. Cell Prolif.

[B44] Theron T, Bohm L (2000). Influence of the G2 cell cycle block abrogator Pentoxifylline on the expression and subcellular location of cyclin B1 and p34 CDC-2 in Hela cervical carcinoma cells. Cell Prolif.

[B45] Theron T, Binder A, Verheye-Dua F, Bohm L (2000). The role of G2 block abrogation, DNA double strand break repair and apoptosis in the radiosensitization of melanoma and squamous cell carcinoma cell lines. Int J Radiat Biol.

[B46] Tolmach LJ, Busse PW (1980). The action of caffeine on X-irradiated Hela cells. IV. Progression delays and enhanced cell killing at high caffeine concentrations. Radiat Res.

[B47] Vernimmen F, Verheye-Dua F, du Toit H, Bohm L (1994). Effect of Pentoxifylline on irradiation damage and tumour growth. Strahlenther Onkol.

[B48] Wang H, Wang X, Iliakis G, Wang Y (2003). Caffeine could not effectively sensitize homologous recombination repair deficient cells to ionizing radiation induced cell killing. Radiat Res.

[B49] Wang X, Wang H, Iliakis G, Wang Y (2003). Caffeine induced radiosensitisation is independent of non-homologous end joining of DNA double strand breaks. Radiat Res.

[B50] Ward A, Clissold SP (1987). Pentoxifylline: A review of the pharmacokinetic properties and therapeutic efficacy. Drugs.

[B51] Zhou BBS, Elledge SJ (2000). The DNA damage response: Putting checkpoints into perspective. Nature.

[B52] Zywietz F, Bohm L, Sagowski C (2004). Pentoxifylline enhances tumour oxygenation and radiosensitivity in rat rhabdomyosarcomas during continuous hyperfractionated irradiation. Strahlenther Onkol.

[B53] Horsman M, Bohm L, Margison GP, Milas L, Rosier JF, Safrany G, Selzer E, Verheij M, Hendry JH (2006). Tumour radiosenstizers – the current status of development of various approaches : Report of an IAEA meeting. Int J Radiat Oncol Biol Phys.

[B54] Bohm L Inhibition of homologous recombination repair with pentoxifylline targets G2 cells generated by radiotherapy. Molecular Radiation Biology/Oncology 6/2005: 30 (abstract).

[B55] Diggle CP, Bentley J, Knowles MA, Kiltie AE (2005). Inhibition of double-strand break non-homologous end-joining by cisplatin adducts in human cell extracts. Nucleic Acids Res.

[B56] Raaphorst GP, Leblanc M, Li LF (2005). A comparison of response to cisplatin, radiation and combined treatment for cells deficient in recombination repair pathways. Anticancer Res.

[B57] Kwon HC, Kim SK, Chung WK, Cho MJ, Kim JS, Moon SR, Park WY, Ahn SJ, Oh YK, Yun HG, Na BS (2000). Effect of Pentoxifylline on radiation response of nonsmall cell lung cancer : a Phase III randomised multicenter trial. Radiother Oncol.

[B58] Johnson FE, Harrison BR, McKirgan LW, Raju PI, Roy TK, Virgo KS (1998). A phase II evaluation of Pentoxifylline combined with radiation in the treatment of brain metastases. Int J Oncol.

[B59] Stewart DJ, Dahrouge S, Agboola O, Girard A (1997). Cranial radiation and concomitant cisplatin and mitomycin-C plus resistance modulators for malignant gliomas. J Neurooncol.

[B60] Amirkhosavri A, Meyer T, Warnes G, Amaya M, Malik Z, Biggerstaff JP, Siddiqui FA, Sheramn P, Francis JL (1998). Pentoxifylline inhibits hypoxia induced upregulation of tumour cell tissues factorand vascular endothelial growth factor. Thromb Haematost.

[B61] Ayenc E, Celikkanat S, Kaymakci M, Aksaray F, Ozdem C (2004). Prophylactic effect of Pentoxifylline on radiotherapy complications: A clinical study. Otolaryngology Head and Neck Surgery.

[B62] Hille A, Christiansen H, Pradier O, Herrmann RM, Siekmeyer B, Weiss E, Hilgers R, Hess CF, Schmidberger H (2005). Effect of Pentoxifylline and Tocopherol on radiation proctitis/enteritis. Strahlenther Onkol.

[B63] Delanian S, Porcher R, Ball-Mekias S, Lefaix JL (2003). Randomized, placebo-controlled trial of combined Pentoxifylline and Tocopherol for regression of superficial radiation induced fibrosis. J Clin Oncol.

[B64] Wlodek D, Banath J, Olive PL (1991). Comparison between pulsed field and constant field gel electrophoresis for measurement of DNA double-strand breaks in irradiated Chinese hamster ovary cells. Intern J Radiat Biol.

[B65] Dikomey E, Dahm-Daphi J, Brammer I, Martensen R, Kaina B (1998). Correlation between cellular radiosensitivity and non-repaired double-strand breaks studied in nine mammalian cell lines. Int J Radiat Biol.

